# Potassium channels as an ionic checkpoint in tumor immunity

**DOI:** 10.3389/fphar.2026.1907228

**Published:** 2026-09-10

**Authors:** Huanle Wang, Yinan Zhang, Sufen Jiang, Xiaojiang Li, Yixin Zheng, Yukun Wang, Yue Xu, Cong Xia, Yan Yan

**Affiliations:** 1 Department of Gastrointestinal Surgery, The Second Hospital of Dalian Medical University, Dalian, China; 2 Department of Clinical Laboratory, The Second Hospital of Dalian Medical University, Dalian, China; 3 Department of Clinical Laboratory, Heze Third People’s Hospital, Heze, China; 4 Department of Clinical Laboratory, Dalian No. 3 People’s Hospital, Dalian, China

**Keywords:** cancer immunotherapy, immunometabolism, ionic checkpoint, potassium channels, tumor immune microenvironment

## Abstract

Potassium (K^+^) channels sustain immune-cell activation by coupling K^+^ flux to membrane potential, Ca^2+^ signaling, migration, and metabolic adaptation. In solid tumors, however, extracellular K^+^ accumulation, adenosine, hypoxia, acidosis, oxidative stress, and lipid stress can disrupt ionic homeostasis and channel function. Here, we define an ionic checkpoint as a context-dependent regulatory state in which K^+^ gradients, channel gating and trafficking, membrane potential, and subcellular channel localization determine whether immune cells cross the electrophysiological thresholds required for Ca^2+^ signaling and effector function. We synthesize evidence across adaptive and innate immunity, emphasizing Kv1.3 and KCa3.1 in T- and natural killer-cell responses and dendritic-cell migration, and Kir and K2P channels in myeloid and NK-cell programs. We further examine plasma-membrane–mitochondrial channel crosstalk, redox regulation, and spatial and temporal heterogeneity in the tumor immune microenvironment. Because the same channel may support lymphocyte function while sustaining tumor-cell survival or immunosuppressive myeloid states, neither universal activation nor universal inhibition is appropriate. Direct channel targeting is constrained by cell and organelle selectivity, whereas upstream tumor-microenvironment-directed strategies, particularly adenosine-axis interventions, are clinically more advanced but have not yet established direct restoration of K^+^ channel-supported immune function. Translation will require functional and spatial biomarkers, precision patient stratification, and cell- or organelle-selective delivery.

## 1 Introduction

The development of immune checkpoint blockade (ICB), which restores antitumor immunity by blocking inhibitory receptor–ligand pathways such as PD-1/PD-L1 and CTLA-4, and adoptive cell therapy (ACT), in which tumor-reactive lymphocytes are isolated, expanded, and/or engineered *ex vivo* before administration to patients, has substantially advanced cancer treatment; nevertheless, clinical benefit remains confined to a subset of patients, and both primary and acquired resistance remain common (Petitprez et al., 2020; Heintzman et al., 2022). Accumulating evidence indicates that tumor immune escape is shaped by multiple features of the tumor immune microenvironment (TIME), including imbalances in immune-cell composition, inhibitory receptor–ligand signaling, stromal barriers, metabolic competition, and other local ecological inputs such as the intratumoral microbiota (Binnewies et al., 2018; Mai et al., 2025; Zou et al., 2025). In many solid tumors, however, effector immune cells are not entirely absent. Instead, they persist as tumor-infiltrating lymphocytes, but their activation, cytotoxic capacity, and ability to sustain responses are restricted to varying degrees, resulting in heterogeneous states of dysfunction or exhaustion (van der Leun et al., 2020; Zheng et al., 2021; Philip and Schietinger, 2022). Changes in classical immune-checkpoint expression or immune-cell abundance alone are therefore insufficient to explain the failure of antitumor immunity.

The TIME also has distinct physicochemical properties. Changes in ion concentrations, acid–base balance, and transmembrane electrochemical gradients continuously reshape immune-cell activation thresholds and effector capacity. K^+^ channels have a central role in this process: K^+^ efflux through Kv1.3 and KCa3.1 helps maintain a negative membrane potential, thereby sustaining the activity of ORAI1-containing Ca^2+^ release-activated Ca^2+^ (CRAC) channels and downstream immune-effector programs (Gawali et al., 2021; Chirra et al., 2022). Conversely, K^+^ released from necrotic tumor cells can increase local extracellular K^+^, disrupt intracellular K^+^ homeostasis, and suppress Akt–mTOR signaling, metabolic adaptation, and cytotoxic function (Eil et al., 2016; Vodnala et al., 2019). Adenosine, hypoxia, acidosis, and oxidative stress can also alter channel gating, membrane trafficking, or pharmacological responsiveness. K^+^ channels therefore represent functional nodes linking physicochemical stress in the TIME to Ca^2+^ signaling and immunometabolism.

Against this background, we conceptualize K^+^ channel-related regulation as a context-dependent ionic checkpoint within the TIME. Here, K^+^ channels refer collectively to potassium-selective channels located in the plasma membrane or intracellular organelles. We use ionic checkpoint to denote a regulatory state in which K^+^ gradients, channel gating and trafficking, membrane potential, and subcellular localization determine whether immune cells cross the electrophysiological thresholds required for Ca^2+^ signaling, migration, metabolic adaptation, and effector function. Unlike classical immune checkpoints, which are initiated principally by inhibitory receptor–ligand interactions, an ionic checkpoint is not a single molecular pair and does not imply that K^+^ channel activity is uniformly immunosuppressive. It also differs from metabolic checkpoints, which are defined primarily by nutrient or metabolite availability and metabolic enzymes, and from mechanotransduction, which converts matrix stiffness, confinement, and force into intracellular signaling. These systems intersect, but the proximal variables of the ionic checkpoint are ion gradients and channel functional state.

**FIGURE 1:**
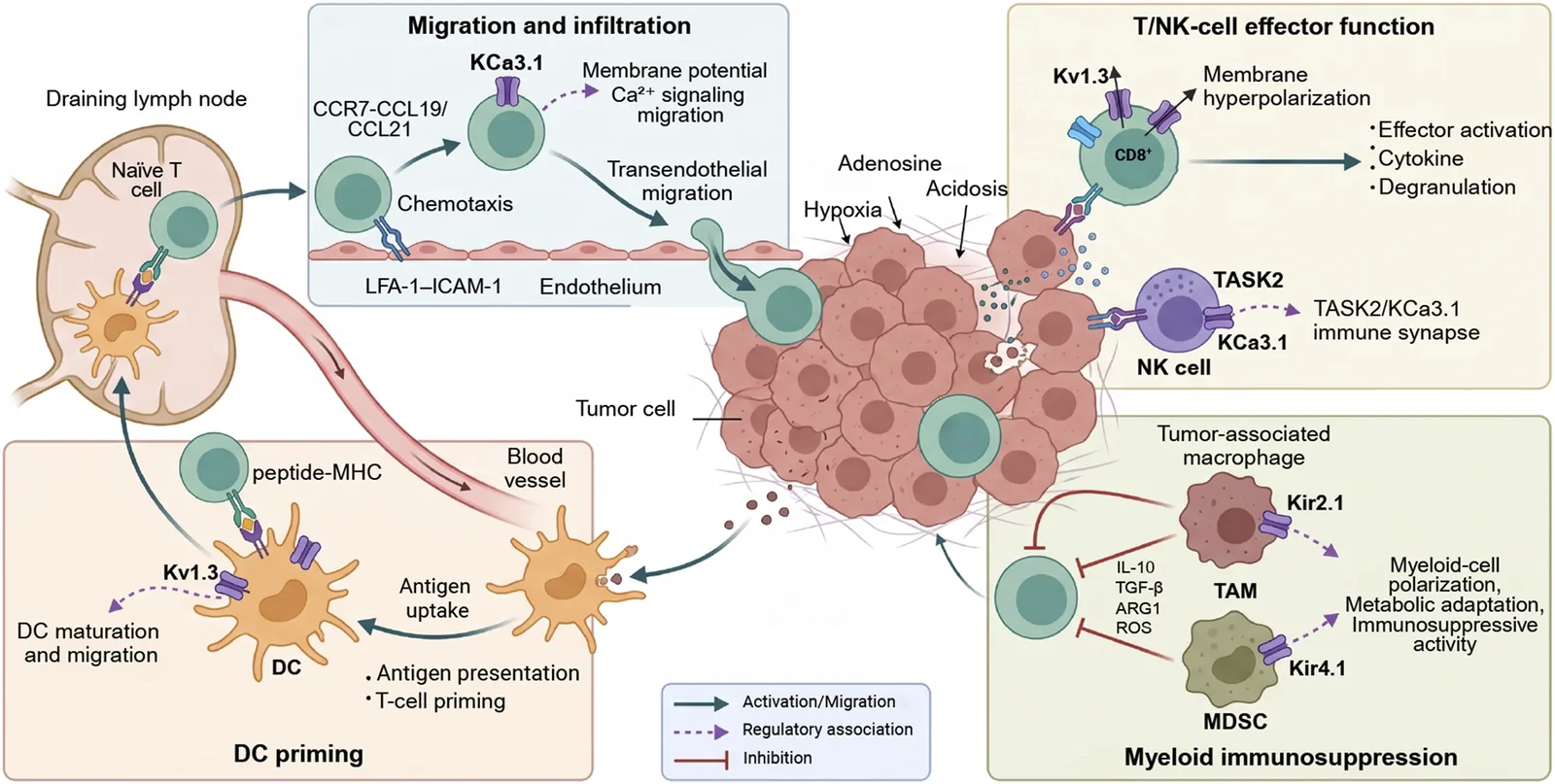
K^+^ channel-dependent regulation of antitumor immunity. K^+^ channels participate in multiple stages of the antitumor immune response, including dendritic-cell antigen uptake and migration, T-cell priming and tumor infiltration, and the cytotoxic functions of T cells and natural killer cells. In parallel, Kir2.1- and Kir4.1-associated metabolic programs support the immunosuppressive activity of tumor-associated macrophages and myeloid-derived suppressor cells, respectively. Tumor microenvironmental factors, including adenosine accumulation, hypoxia and acidosis, further disrupt channel-supported immune functions. Arrows and blunt-ended lines indicate stimulatory and inhibitory effects, respectively.

Focusing primarily on solid tumors, this review systematically examines the roles of K^+^ channels in adaptive and innate antitumor immunity and integrates the effects of TIME stress, Ca^2+^–mitochondrial coupling, reactive oxygen species (ROS), and immunometabolic reprogramming. We then compare the levels of evidence, translational progress, and principal limitations of direct channel targeting and upstream TIME-directed interventions, and assess the potential of K^+^ channels as functional biomarkers and combination-therapy targets. K^+^ channel activity spans dendritic-cell (DC)-mediated antigen uptake and T-cell priming, effector-cell migration and tumor infiltration, T- and natural killer (NK)-cell function, and immunosuppressive reprogramming of myeloid cells, with pronounced cell-type- and spatial-context dependence (Figure 1).

## 2 K^+^ channels in adaptive antitumor immunity

### 2.1 T-cell activation, the immunological synapse, and cytotoxicity

Following recognition of peptide–major histocompatibility complex by the T-cell receptor (TCR), phospholipase Cγ1-mediated production of inositol 1,4,5-trisphosphate triggers Ca^2+^ release from the endoplasmic reticulum. This activates Ca^2+^ release-activated Ca^2+^ channels formed by STIM1 and ORAI1 and initiates sustained store-operated Ca^2+^ entry. Ca^2+^ influx depolarizes the plasma membrane and thereby diminishes its own electrochemical driving force. K^+^ efflux through Kv1.3 and KCa3.1 preserves a negative membrane potential, sustains Ca^2+^ signaling, and supports activation of the calcineurin–nuclear factor of activated T cells pathway, thereby promoting interleukin-2 expression, clonal expansion, and effector differentiation (Vaeth et al., 2020; Chirra et al., 2022). The relative contributions of the two channels vary with T-cell differentiation state. Activated naïve and central memory T cells predominantly upregulate KCa3.1, whereas effector memory T cells exhibit greater dependence on Kv1.3. Channel abundance alone therefore cannot define the functional state of different T-cell subsets.

The immunological synapse provides a spatially organized platform for TCR signaling, cytoskeletal remodeling, and local ion transport, within which Kv1.3 accumulates at the cytotoxic T-cell–target-cell interface and KCa3.1 redistributes after antigen stimulation (Panyi et al., 2004; Nicolaou et al., 2007). Restricting the lateral movement of Kv1.3 toward the immunological synapse alters antigen-induced Ca^2+^ responses without affecting Ca^2+^ signals elicited by nonsynaptic TCR stimulation, demonstrating that subcellular localization is as important as channel abundance (Nicolaou et al., 2009). In primary human CD4^+^ T cells, Kv1.3 initially localizes to the F-actin-rich distal pole of the synapse and subsequently moves toward the synaptic center before internalization, further illustrating its dynamic organization during synapse formation and maturation (Capera et al., 2024).

Ca^2+^ signaling also regulates lytic-granule transport, membrane fusion, and exocytosis in cytotoxic T cells. Effective killing, however, depends on an optimal Ca^2+^ range rather than an unrestricted increase in intracellular Ca^2+^ (Kaschek et al., 2021). In patients with head and neck cancer, CD8^+^ tumor-infiltrating lymphocytes exhibit reduced Kv1.3 expression and channel activity together with attenuated Ca^2+^ responses. Cells with higher Kv1.3 expression show greater proliferative capacity and granzyme B expression, suggesting that intact Kv1.3 function is associated with the effector competence of tumor-infiltrating T cells (Chimote et al., 2017). By maintaining membrane potential and local Ca^2+^ signaling, Kv1.3 and KCa3.1 provide the electrophysiological conditions required for T-cell activation and target-cell killing, rather than directly executing cytotoxicity.

### 2.2 Migration, tumor infiltration, and spatial localization

To establish effective contact with tumor cells, circulating effector T cells must successively undergo chemotactic recruitment, endothelial adhesion and transendothelial migration, interstitial motility, and retention within the tumor parenchyma. The detection of CD8^+^ T cells in tumor tissue therefore does not necessarily indicate effective infiltration. T cells that accumulate around vessels or within the tumor stroma but fail to enter the tumor parenchyma remain characteristic of an immune-excluded state (Tiwari et al., 2023). K^+^ channels do not directly determine chemotactic direction; instead, by regulating membrane potential, Ca^2+^ signaling, cell volume, and cytoskeletal dynamics, they provide electrophysiological support for T-cell polarization and sustained migration (Chirra et al., 2022).

The contribution of KCa3.1 to T-cell migration is spatially specific. In activated human T cells migrating on ICAM-1-coated surfaces, KCa3.1 and TRPM7 predominantly localize to the uropod, whereas Kv1.3 and CRAC channels are concentrated at the leading edge. Blocking KCa3.1 or TRPM7 reduces both the number and velocity of migrating cells, whereas Kv1.3 blockade has no comparable effect in this model, indicating that these channels have nonredundant migratory functions (Kuras et al., 2012). Among human effector memory CD8^+^ T cells, IL-7Rαlow cells with low KCa3.1 activity display impaired motility and transendothelial migration in the presence of ICAM-1 and CXCL12. Restoration of KCa3.1 activity by interleukin-2 or interleukin-15 improves chemotaxis, further supporting a functional relationship between KCa3.1 and tissue-homing capacity (Sim et al., 2017).

Activated CD8^+^ T cells from patients with head and neck squamous cell carcinoma (HNSCC) exhibit reduced KCa3.1 activity without a corresponding decrease in channel expression. In the presence of adenosine, their migration toward CXCL12 is more profoundly impaired, whereas the KCa3.1 activators 1-EBIO and NS309 partially restore chemotaxis (Chimote et al., 2018). These findings, however, derive primarily from *in vitro* migration assays and do not directly demonstrate that KCa3.1 enhancement improves tumor-parenchymal infiltration in patients. Histological analyses further show that CD8^+^ tumor-infiltrating lymphocytes with high Kv1.3 expression, Ki67 abundance, and granzyme B levels are largely retained in the tumor stroma rather than entering the tumor epithelial compartment (Chimote et al., 2017). In the CT26 colon cancer model, tumor uptake of a Kv1.3-targeted tracer was associated with the accumulation of Kv1.3^+^ CD8^+^ effector memory T cells and response to immunotherapy (Goggi et al., 2022). Intact K^+^ channel function can therefore support T-cell migration and tissue homing, but current evidence is insufficient to establish that enhancing Kv1.3 or KCa3.1 alone improves tumor-parenchymal infiltration. Final T-cell localization remains jointly constrained by chemokines, tumor vasculature, the extracellular matrix, and stromal barriers.

### 2.3 T-cell dysfunction and interactions with classical immune checkpoints

The relationship between K^+^ homeostasis and T-cell dysfunction is not unidirectional; it depends on intracellular K^+^ concentrations and the intensity of channel-supported Ca^2+^ signaling. Loss of *Atp1a1* lowers intracellular K^+^, causes ROS accumulation and sustained basal signaling, increases expression of exhaustion-associated receptors, and compromises the intratumoral persistence and antitumor activity of CD8^+^ T cells. K^+^ supplementation or antioxidant treatment partially reverses this phenotype (Collier et al., 2024). Limited methionine availability during early TCR activation reduces methylation of KCa3.1 at Arg350, enhances KCa3.1-associated Ca^2+^ influx and NFAT1 nuclear translocation, and ultimately drives T-cell dysfunction. Disruption of this methylation site increases the exhausted phenotype in murine tumor models, whereas early methionine supplementation reduces NFAT1 activity in tumor-infiltrating T cells and improves both antitumor activity and the response to PD-1 blockade (Sharma et al., 2025). Thus, both intracellular K^+^ deficiency and persistently excessive Ca^2+^–NFAT1 signaling can promote T-cell exhaustion, indicating that K^+^ channel function must be maintained within an appropriate range.

Elevated extracellular K^+^ also directly links disrupted ionic homeostasis to classical immune-checkpoint expression. In activated human peripheral-blood T cells, high K^+^ suppresses Akt–mTOR signaling, cytokine production, and tumor-cell killing while increasing PD-1 protein expression. Selective KCa3.1 activation partially restores IFN-γ production, whereas high K^+^ does not substantially alter total Kv1.3 or KCa3.1 expression, suggesting that the effect primarily reflects changes in the transmembrane K^+^ gradient and channel functional state (Ong et al., 2019). A recent murine melanoma study further showed that increased intratumoral K^+^ inhibits CD8^+^ T-cell proliferation, activation, and cytotoxicity without reducing cell numbers and is accompanied by impaired glucose uptake, glycolysis, and mitochondrial fitness. Local reduction of interstitial K^+^ with a K^+^-scavenging hydrogel restored CD8^+^ T-cell metabolic and effector function, attenuated exhaustion, and enhanced the antitumor activity of adoptive cell therapy (Liu et al., 2026).

PD-1/PD-L1 signaling can, in turn, regulate K^+^ channel function. PD-L1 stimulation reduces KCa3.1 activity and Ca^2+^ signaling in CD8^+^ T cells, whereas PD-1 or PD-L1 blockade restores KCa3.1 and Kv1.3 currents in CD8^+^ T cells from patients with HNSCC. Intracellular supplementation with phosphatidylinositol 3-phosphate reverses PD-L1-mediated KCa3.1 inhibition, supporting a PI3K–PI3P-related regulatory mechanism. Channel protein expression does not change in parallel, indicating that checkpoint blockade primarily restores channel function (Gawali et al., 2021). In patients receiving neoadjuvant pembrolizumab, recovery of K^+^ channel activity is accompanied by improved Ca^2+^ signaling and chemotactic capacity, with the increase in Kv1.3 activity being more pronounced among clinical responders (Newton et al., 2020). Disrupted K^+^ homeostasis can therefore promote PD-1 expression, whereas PD-1/PD-L1 signaling can suppress K^+^ channel function, creating a mutually reinforcing inhibitory loop. This relationship is currently supported mainly by studies of the PD-1/PD-L1 axis in HNSCC; direct evidence that CTLA-4, LAG-3, TIM-3, or TIGIT exerts comparable channel-regulatory effects remains lacking.

## 3 K^+^ channels in innate antitumor immunity

### 3.1 Dendritic-cell antigen processing, migration, and T-cell priming

Antitumor T-cell responses begin with tumor-antigen uptake, processing, and presentation by DCs. Conventional type 1 DCs have strong cross-presenting capacity and can present exogenous tumor antigens on major histocompatibility complex class I molecules to prime naïve CD8^+^ T cells. In brain tumor models, cDC1 cells not only participate in local antigen uptake but also transport tumor antigens to the cervical draining lymph nodes; their absence markedly impairs tumor-specific CD8^+^ T-cell priming and antitumor immunity (Bowman-Kirigin et al., 2023). The spatial role of DCs is not restricted to unidirectional migration from tumors to lymph nodes. A subset of activated CCR7^+^ DCs can be retained within tumors and sustain cytotoxic immunity through local antigen presentation and interactions with other immune cells (Lee et al., 2024). DC-mediated antitumor immunity therefore depends on both antigen-processing capacity and appropriate distribution between tumor and lymphoid tissues.

K^+^ channels may provide essential electrophysiological support during the early stages of antigen processing. DC-specific loss of Dlg1 destabilizes voltage-gated K^+^ channels, including Kv1.3, reduces stimulus-induced K^+^ efflux and subsequent Ca^2+^ influx, and impairs antigen endocytosis, phagocytosis, and proinflammatory cytokine production (Dong et al., 2019). The functional relationship between KCa3.1 and DC migration is comparatively well established. CCL19 and CCL21 elicit CCR7-dependent Ca^2+^ signals and activate KCa3.1; channel-mediated K^+^ efflux helps maintain membrane potential and sustained Ca^2+^ entry. In murine lung DCs, pharmacological blockade or silencing of KCa3.1 reduces CCL19-and CCL21-induced migration and lymph-node recruitment (Shao et al., 2015). In immature human monocyte-derived DCs, KCa3.1 activation enhances Ca^2+^ signaling and migration, whereas channel inhibition reduces chemotaxis and alters CCR7 expression (Crottès et al., 2016). Nevertheless, the causal roles of Kv1.3 and KCa3.1 as permissive regulators of antigen uptake and migration in tumor-associated DCs require validation in cell-subset-specific animal models.

### 3.2 NK-cell cytotoxicity

NK-cell killing proceeds through target-cell engagement, immunological-synapse formation, cytotoxic-granule polarization, and exocytosis. Early electrophysiological and pharmacological studies found that inhibition of K^+^ currents interfered with distinct stages of human NK-cell killing, providing the first functional link between K^+^ channels and NK-cell cytotoxicity (Schlichter et al., 1986). K^+^ efflux maintains a negative membrane potential and thereby supports sustained Ca^2+^ entry. ORAI1 deficiency markedly impairs degranulation and target-cell lysis by cytotoxic lymphocytes (Maul-Pavicic et al., 2011). The relationship between Ca^2+^ signaling and killing efficiency, however, is not simply linear. Excessive Ca^2+^ signaling can promote granule release but may disrupt granule allocation and reduce serial-killing efficiency (Li Y. et al., 2022).

Human NK cells predominantly express Kv1.3, KCa3.1, and TASK2, and their functional requirements vary with subset and activation state. TASK2 contributes to NK-cell proliferation, LFA-1-mediated target-cell adhesion, and cytotoxic-granule release. TASK2 inhibition markedly reduces degranulation in resting NK cells, whereas IL-15 activation partially alleviates this dependence, indicating that cytokine stimulation reshapes K^+^ channel requirements (Schulte-Mecklenbeck et al., 2015). The effect of KCa3.1 on NK-cell killing also depends on Ca^2+^ signal strength and tumor model. The plant-derived small molecule LJ101019C increases Kv1.3 current and protein expression in NK cells, enhances AKT/mTOR signaling, and improves killing of MIA PaCa-2 pancreatic cancer cells (Geng et al., 2020). Patient-derived NK cells exhibit impaired KCa3.1 function and chemotaxis, whereas KCa3.1 activation enhances their migration, release of cytotoxic mediators, and killing of Cal27 tumor spheroids. In humanized tumor-bearing mice, SKA-31 increases the intratumoral accumulation of total and granzyme B-positive NK cells and reduces tumor burden (Alshwimi et al., 2025). Not all NK-cell K^+^ channels directly regulate cytotoxicity. A recent study showed that NK-cell-specific deletion of *Kcnj8* reduces mature CD27^−^CD11b^+^ and KLRG1^+^ NK-cell populations without affecting degranulation against tumor cells or short-term tumor-cell clearance *in vivo* (Samper et al., 2024).

### 3.3 Myeloid-cell reprogramming and immunosuppression

The functional state of tumor-associated macrophages (TAMs) is closely linked to their metabolic program, and K^+^ channels can translate local ionic changes into metabolic and phenotypic reprogramming. High interstitial K^+^ limits the antitumor plasticity of TAMs through Kir2.1. Conditional deletion of *Kcnj2* in myeloid cells or pharmacological inhibition of Kir2.1 reduces electrochemical-gradient-dependent glutamine uptake, shifts metabolism from oxidative phosphorylation toward glycolysis, and promotes an inflammatory, antitumor TAM state. Kir2.1 blockade also suppresses the growth of syngeneic murine tumors and patient-derived xenografts (Chen et al., 2022).

KCa3.1 also contributes to the maintenance of myeloid-cell states in glioma. In the GL261 model, the KCa3.1 inhibitor TRAM-34 reduces expression of protumor genes and shifts infiltrating microglia and macrophages toward a more inflammatory, antitumor phenotype (Grimaldi et al., 2016). Intravital two-photon imaging further showed that KCa3.1 inhibition rapidly reduces the hypertrophic morphology of myeloid cells within the tumor core, increases process ramification, and is accompanied by reduced cellular density in this region (Massenzio et al., 2022). Because KCa3.1 is expressed by both glioma and myeloid cells, however, systemic TRAM-34 treatment cannot fully distinguish their respective contributions. Another study found that trabectedin does not directly block Kv1.3 or Kv1.5, but increases macrophage Kv1.3 currents and alters the relative composition of Kv and KCa currents in TAMs (Peraza et al., 2023).

High K^+^ also supports metabolic adaptation in myeloid-derived suppressor cells (MDSCs) by inducing autophagy and increasing mitochondrial respiration (Thylur Puttalingaiah et al., 2024). *In vitro* and *ex vivo* exposure to excess KCl increases LC3β expression, mitochondrial respiration, and AKT and ERK phosphorylation. Despite reduced Arg1 and iNOS expression, MDSCs retain their capacity to suppress T-cell proliferation, whereas autophagy inhibition reverses the metabolic changes. A recent study further showed that high K^+^ upregulates Kcnj10 and its encoded channel Kir4.1 in MDSCs. Kir4.1 increases FABP3 expression, promotes fatty-acid uptake and oxidation, and thereby enhances MDSC-mediated suppression of CD8^+^ T-cell proliferation and IFN-γ production. Pharmacological Kir4.1 inhibition attenuates MDSC-mediated immunosuppression and improves the response of murine tumors to anti-PD-1 treatment (Xie et al., 2026). The tumor-related functions of other myeloid K^+^ channels remain undefined.

## 4 Remodeling of the ionic checkpoint by the tumor microenvironment

### 4.1 Extracellular K^+^ accumulation and adenosine-mediated suppression

Tumor necrosis releases high concentrations of intracellular K^+^ into the interstitium, increasing local extracellular K^+^ levels. Although this change thermodynamically reduces the driving force for K^+^ efflux, direct evidence indicates that T-cell suppression is not mediated primarily by changes in membrane potential. Elevated extracellular K^+^ causes intracellular K^+^ accumulation and inhibits TCR-induced Akt and mTOR phosphorylation through a PP2A-dependent mechanism, thereby reducing effector-molecule expression. Accordingly, Kv1.3 overexpression enhances K^+^ efflux, lowers intracellular K^+^, improves the effector function of tumor-specific T cells, and enhances tumor control in tumor-bearing mice (Eil et al., 2016).

A high-K^+^ environment can also influence T-cell fate through metabolic and epigenetic mechanisms. Elevated extracellular K^+^ restricts nutrient uptake, induces autophagy, and reduces histone acetylation at loci associated with effector function and exhaustion. This process suppresses immediate effector activity while preserving T-cell stemness, multipotency, and persistence *in vivo* (Vodnala et al., 2019). The relationship is therefore not a simple linear inhibition. When *Atp1a1* loss decreases intracellular K^+^, CD8^+^ T cells develop impaired Ca^2+^ entry, ROS accumulation, sustained signaling, and an exhausted phenotype; K^+^ supplementation or antioxidant treatment partially reverses these changes (Collier et al., 2024). Maintaining intracellular K^+^ within an appropriate range is thus more important than simply increasing K^+^ efflux.

Adenosine constitutes a distinct microenvironmental suppressive pathway that acts on K^+^ channels. Extracellular ATP is sequentially hydrolyzed by CD39 and CD73 to generate adenosine, which predominantly activates cAMP and PKA-I signaling through the A_2_A receptor (Deaglio et al., 2007). In activated human T cells, adenosine selectively inhibits KCa3.1 without substantially affecting Kv1.3 or TRPM7. KCa3.1 inhibition reduces T-cell migration on ICAM-1 and decreases IL-2 secretion, demonstrating that receptor-coupled adenosine signaling can directly impair channel-supported immune function (Chimote et al., 2013).

Compared with healthy donors, peripheral-blood CD8^+^ T cells from patients with HNSCC have lower KCa3.1 activity despite no corresponding reduction in channel expression or downstream A_2_A receptor signaling. This functional defect increases sensitivity to adenosine, whereas KCa3.1 activators restore chemotaxis in adenosine-rich three-dimensional environments (Chimote et al., 2018). Subsequent work linked the defect to reduced membrane-proximal calmodulin and impaired calmodulin–KCa3.1 binding (Chimote et al., 2020). Extracellular K^+^ accumulation and adenosine are therefore not redundant suppressive mechanisms: the former primarily alters intracellular K^+^ homeostasis and metabolic and epigenetic programs, whereas the latter selectively restricts KCa3.1 through A_2_A receptor signaling. Together, these processes reduce the immediate responsiveness and tumor-infiltrating capacity of effector lymphocytes.

### 4.2 Hypoxia and acidosis

Hypoxic regulation of Kv1.3 is time dependent. Acute hypoxia suppresses approximately 20% of the Kv1.3 current in freshly isolated human T lymphocytes and Jurkat T cells within minutes, indicating that this early response predominantly reflects altered channel function rather than reduced expression (Conforti et al., 2003). In primary human CD3^+^ T cells, hypoxia-induced Kv1.3 inhibition causes membrane depolarization and attenuates the Ca^2+^ response to CD3/CD28 stimulation in a subset of cells. No direct effect on KCa3.1 or CRAC channels was observed, further supporting a predominant role for Kv1.3 in the acute response (Robbins et al., 2005).

Prolonged hypoxia primarily affects intracellular trafficking and surface availability of Kv1.3. Exposure of Jurkat T cells to 1% O_2_ for 24 h reduces Kv1.3 protein abundance and K^+^ current density without a corresponding change in *KCNA3* mRNA (Conforti et al., 2003). Further studies showed that chronic hypoxia downregulates the adaptor protein complex AP-1, disrupts clathrin-mediated anterograde vesicular trafficking of Kv1.3, retains the channel in the trans-Golgi network, and reduces both plasma-membrane expression and functional current (Chimote et al., 2012). *KCNA3* transcription, total Kv1.3 protein, plasma-membrane channel abundance, and K^+^ current therefore cannot be used interchangeably. Because existing studies predominantly used peripheral-blood T cells and Jurkat cells, whether the same trafficking defect occurs in tumor-infiltrating T cells requires direct validation.

An acidic environment can independently suppress antitumor immunity. A pH of 6.0–6.5 reduces cytotoxic activity and cytokine secretion by human and murine tumor-specific CD8^+^ T cells and attenuates TCR-proximal STAT5 and ERK signaling; restoration of extracellular pH to the physiological range reverses these changes (Calcinotto et al., 2012). The direct effect of acidity on Kv1.3, however, cannot be described simply as channel inhibition. Studies in heterologous expression systems showed that lowering extracellular pH alters the kinetics of Kv1.3 slow inactivation and, at physiological ionic strength, delays rather than accelerates channel inactivation (Somodi et al., 2004).

Patch-clamp experiments in activated human peripheral-blood lymphocytes and KCa3.1-expressing CHO cells showed that extracellular pH values of 6.0–8.0 had little effect on basal KCa3.1 currents or on the immediate activating effects of riluzole or SKA-31. By contrast, lowering intracellular pH to 6.5 caused a progressive, poorly reversible decline in the efficacy of both activators (Cozzolino and Panyi, 2024). In murine tumor models, limiting T-cell intracellular acidification by inhibiting anion exchanger 2 or enhancing expression of the proton channel HVCN1 improves the antitumor activity of tumor-specific T cells and chimeric antigen receptor T (CAR-T) cells, although this effect has not been directly attributed to KCa3.1 (Navarro et al., 2022). Hypoxia and acidity may therefore jointly impair channel-supported immune function and reduce the efficacy of some KCa3.1 activators, but direct evidence for their combined effects in tumor-infiltrating immune cells remains limited.

### 4.3 K^+^ channel–mitochondrial crosstalk under TIME stress

#### 4.3.1 Plasma-membrane K^+^ channels, Ca^2+^ entry, and mitochondrial feedback

Plasma-membrane Kv1.3 and KCa3.1 couple ionic signaling to mitochondrial function by preserving the electrochemical driving force for Ca^2+^ entry. After TCR activation, K^+^ efflux through these channels maintains a relatively negative membrane potential; inhibition of KCa3.1 or loss of Kv1.3 function depolarizes the membrane, shortens sustained CRAC-channel activity, and reduces cytokine secretion and other effector responses (Fanger et al., 2001; Hou et al., 2014). Mitochondria positioned near CRAC channels then take up Ca^2+^ through the mitochondrial calcium uniporter complex in a mitochondrial membrane potential (ΔΨm)-dependent manner. This uptake buffers subplasmalemmal Ca^2+^ microdomains, limits Ca^2+^-dependent CRAC inactivation, and supports NFAT nuclear translocation. Dissipation of ΔΨm or inhibition of mitochondrial Ca^2+^ uptake therefore feeds back to reduce store-operated Ca^2+^ entry (Hoth et al., 2000). During immunological-synapse formation, mitochondrial redistribution toward the synapse further sustains local Ca^2+^ signals (Quintana et al., 2007).

Matrix Ca^2+^ can stimulate tricarboxylic-acid-cycle dehydrogenases and ATP production, linking channel-supported Ca^2+^ entry to metabolic output. In primary human CD4^+^ T cells, TCR stimulation rapidly increases mitochondrial Ca^2+^, and mitochondrial calcium uniporter (MCU) complex knockdown reduces respiration, ATP production, migration, and cytokine secretion (Shumanska et al., 2025). By contrast, T-cell-specific Mcu deletion in mice does not substantially impair respiration or immune function, indicating that chronic genetic loss can be compensated and that MCU dependence varies with species, differentiation state, and experimental design (Wu et al., 2021). TIME stress further weakens this coupling. Tumor-infiltrating T cells exhibit reduced mitochondrial mass and respiratory capacity, and persistent antigen stimulation under hypoxia promotes mtROS accumulation and exhaustion (Scharping et al., 2016; Scharping et al., 2021). Accumulation of depolarized mitochondria during defective mitophagy is likewise associated with terminal CD8^+^ T-cell exhaustion (Yu et al., 2020). Loss of ΔΨm, ATP deficiency, and defective Ca^2+^ buffering can therefore reduce Na^+^/K^+^-ATPase support, channel trafficking, and sustained Ca^2+^ signaling, although direct channel-specific causality remains incompletely defined.

#### 4.3.2 Mitochondrial K^+^ channel pools and compartment-specific effects

K^+^ channels located in mitochondria must be distinguished from their plasma-membrane counterparts. Plasma-membrane Kv1.3 and KCa3.1 support membrane polarization, Ca^2+^ entry, and migration in effector lymphocytes, whereas mitochondrial pools participate in regulation of ΔΨm, matrix volume, ROS production, and apoptosis. Mitochondria-targeted PAPTP and PCARBTP inhibit mitoKv1.3 and disrupt mitochondrial function in pancreatic ductal adenocarcinoma models, while mitoTRAM-34 and rev-mitoTRAM target mitoKCa3.1, increase mtROS, reduce ΔΨm, fragment the mitochondrial network, and induce tumor-cell death (Bachmann et al., 2022; Li W. et al., 2022). These studies establish organelle-specific pharmacology but primarily assess tumor-cell endpoints. They do not demonstrate that inhibition of mitochondrial Kv1.3 or KCa3.1 restores antitumor immune function.

This compartment distinction has direct experimental and therapeutic implications. A compound reaching both the plasma membrane and mitochondria may suppress a channel pool required for lymphocyte activation while inhibiting a mitochondrial pool required for tumor-cell survival. Acute electrophysiological blockade, long-term genetic deletion, altered channel trafficking, and mitochondria-targeted inhibition are therefore not interchangeable interventions. Studies should measure plasma-membrane current, surface density, and Ca^2+^ signaling separately from ΔΨm, mitochondrial K^+^ flux, mtROS, and apoptosis, and should manipulate tumor cells and immune cells independently before testing cocultures. Without this compartment-aware design, a net change in tumor growth cannot identify whether the dominant mechanism is tumor-cell killing, immune-cell modulation, or both.

#### 4.3.3 ROS-dependent channel regulation and metabolic consequences

ROS can modify K^+^ channel gating and abundance through several mechanisms. Oxidation of cysteine and methionine residues can alter local electrostatics, pore conformation, and inactivation kinetics; early electrophysiological studies demonstrated redox-dependent changes in A-type and Shaker K^+^ channels (Ruppersberg et al., 1991; Chen et al., 2000). In Jurkat T cells, Fe_2_O_3_ nanoparticles reduce Kv1.3 current density and alter inactivation and recovery in a manner partially reversed by intracellular NADPH, implicating redox regulation of the Kvβ2 auxiliary subunit (Yan et al., 2015). Sustained oxidation can also change surface availability: Cys-SOH formation in Kv1.5 promotes internalization, impairs recycling, and facilitates degradation (Svoboda et al., 2012). ROS effects are not uniform; in endothelial cells, an H_2_O_2_ donor increases KCa3.1 expression and current, whereas a superoxide donor has the opposite effect (Choi et al., 2013). ROS species, concentration, duration, auxiliary subunits, and cell type must therefore be reported when interpreting channel function.

**FIGURE 2:**
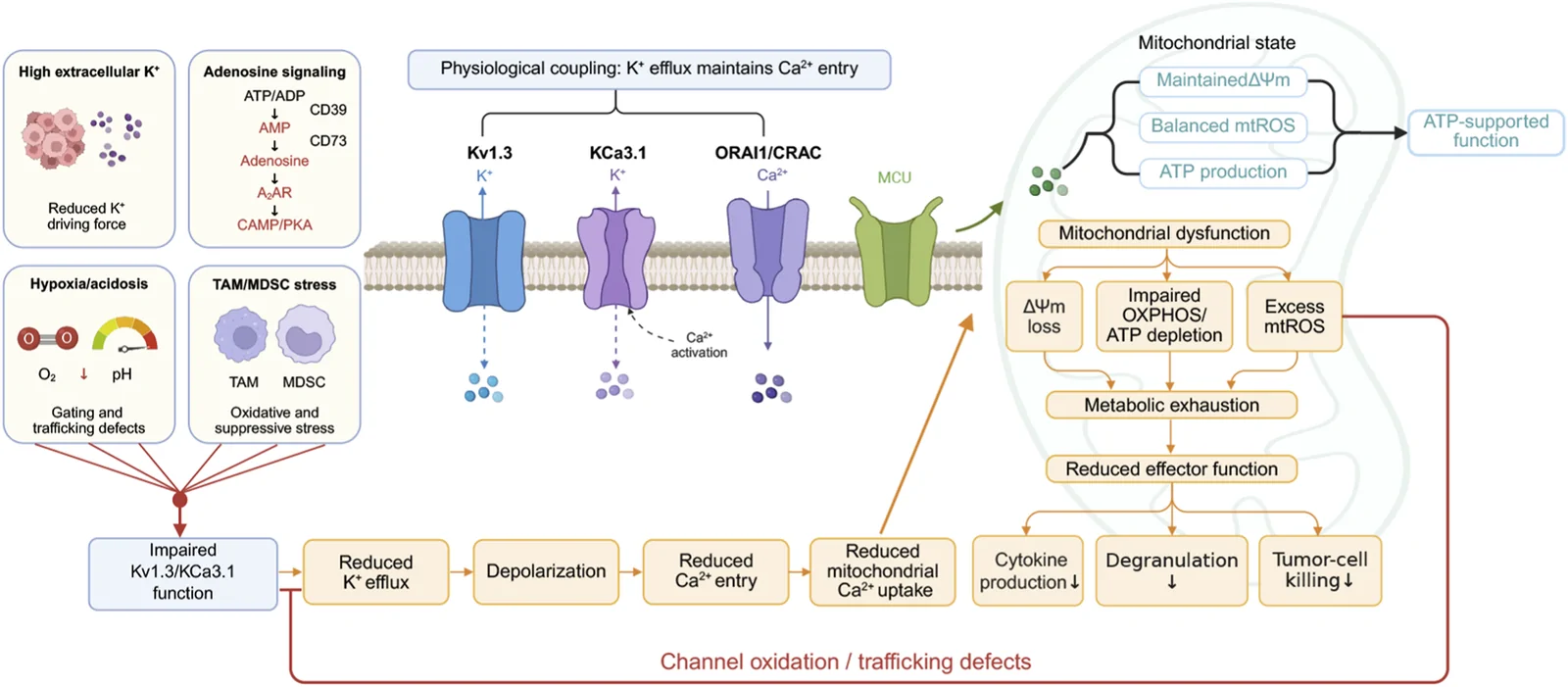
K^+^ channel–Ca^2+^–mitochondrial coupling under tumor immune microenvironmental stress. Under physiological conditions, K^+^ efflux through Kv1.3 and KCa3.1 helps maintain the negative membrane potential required for ORAI1/CRAC-mediated Ca^2+^ entry. Mitochondrial Ca^2+^ uptake subsequently supports mitochondrial membrane potential, oxidative phosphorylation and ATP production, thereby sustaining immune-effector functions. Within the tumor immune microenvironment, elevated extracellular K^+^, adenosine, hypoxia, acidosis, and oxidative stress act through partially distinct mechanisms to disrupt intracellular K^+^ homeostasis, channel-supported Ca^2+^ signaling, and metabolic programs. These changes converge on mitochondrial dysfunction, excessive mitochondrial reactive oxygen species production, and immune-cell exhaustion. Oxidative damage may further impair channel function, forming a self-reinforcing cycle of metabolic and functional deterioration.

Redox signaling also has a biphasic relationship with immune function. A spatially and temporally restricted complex III-derived mtROS pulse contributes to NFAT activation, IL-2 expression, and antigen-specific T-cell expansion, whereas persistent mtROS accumulation during chronic stimulation or hypoxia promotes mitochondrial depolarization and dysfunction (Sena et al., 2013; Scharping et al., 2021). Oxidative stress suppresses cytokine release, degranulation, and tumor-cell killing in human tumor-infiltrating lymphocytes, NK cells, and CAR-T cells, and enhancement of NRF2-dependent antioxidant responses partially preserves these functions (Renken et al., 2022). CD36-dependent uptake of oxidized lipids can also increase lipid peroxidation and impair CD8^+^ T-cell function (Ma et al., 2021), but direct evidence that lipid peroxidation alters Kv1.3 or KCa3.1 synaptic recruitment remains lacking. K^+^ channels may influence glycolysis, oxidative phosphorylation, lipid use, and glutamine metabolism indirectly through membrane potential and Ca^2+^ signaling; however, direct causal evidence linking individual channel subtypes to these metabolic programs or to mitophagy remains uneven, even though mitochondrial quality control itself affects T-cell exhaustion (Yu et al., 2020; Zhang et al., 2025). The most defensible model is therefore a bidirectional K^+^ channel–Ca^2+^–mitochondrial–ROS loop rather than a linear pathway (Figure 2).

### 4.4 Spatial and temporal heterogeneity of ionic checkpoints

An ionic checkpoint is unlikely to be uniform across a solid tumor. Necrotic regions can accumulate K^+^ released from damaged tumor cells, whereas hypoxic and acidic regions impose additional constraints on channel gating, intracellular pH, Ca^2+^ signaling, mitochondrial fitness, and pharmacological responsiveness. Perivascular and invasive-margin regions may have relatively greater oxygen and nutrient availability but remain limited by abnormal endothelium, extracellular-matrix barriers, or immune exclusion. These regional differences are accompanied by variation in the abundance and functional states of CD8^+^ T cells, NK cells, DCs, TAMs, and MDSCs. Consequently, the same channel may support effector-lymphocyte activity in one niche while sustaining tumor-cell survival or immunosuppressive myeloid metabolism in another (Eil et al., 2016; Chimote et al., 2017; Chen et al., 2022; Xie et al., 2026).

Ionic-checkpoint states are also temporally dynamic. Tumor growth, vascular remodeling, necrosis, and changes in immune-cell composition can progressively alter local K^+^, oxygenation, acidity, adenosine production, and oxidative stress. Radiotherapy and cytotoxic therapy can transiently increase tumor-cell death and extracellular ATP release, which may subsequently be converted to immunosuppressive adenosine through the CD39–CD73 pathway. Immunotherapy also changes channel function; PD-1 blockade increases Kv1.3 and KCa3.1 currents in cytotoxic T cells from patients with HNSCC, although recovery varies between patients and is more pronounced for Kv1.3 among clinical responders (Newton et al., 2020; Gawali et al., 2021). A single pretreatment biopsy therefore cannot represent ionic-checkpoint activity across all tumor regions or treatment stages. At present, no study has simultaneously mapped local extracellular K^+^ concentration, channel function, immune-cell identity, and spatial position within the same human tumor.

## 5 Therapeutic targeting and clinical translation

**FIGURE 3:**
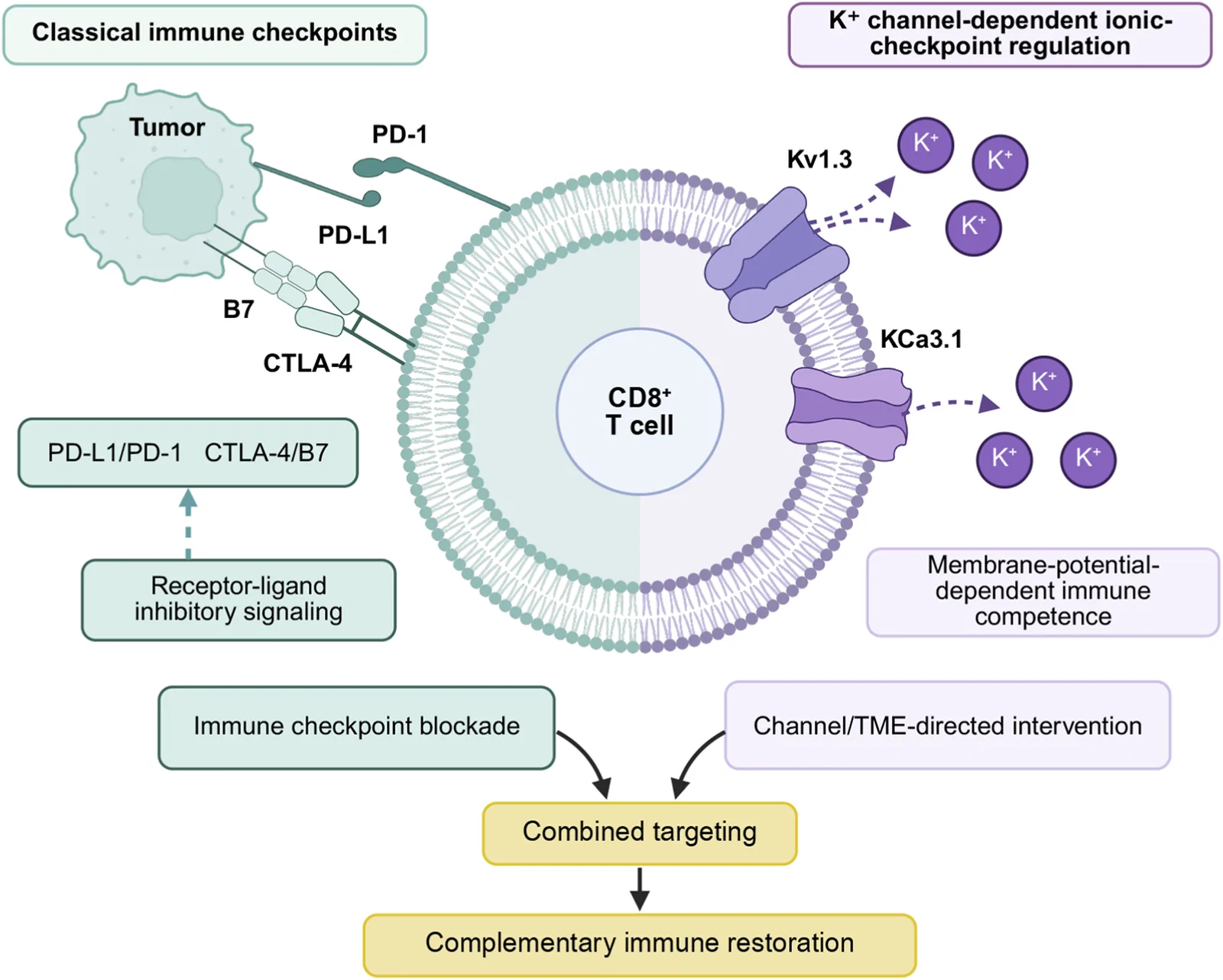
Comparison of classical immune checkpoints and K^+^ channel-dependent ionic-checkpoint regulation. Classical immune checkpoints, represented by PD-1/PD-L1 and CTLA-4/B7, suppress T-cell activation through inhibitory receptor–ligand interactions. By contrast, K^+^ channel-dependent ionic-checkpoint regulation involves membrane-potential control, K^+^ homeostasis and channel-supported Ca^2+^ signaling, which collectively influence immune-cell activation, metabolism and effector function. These two regulatory layers may provide complementary therapeutic targets for restoring antitumor immunity.

**FIGURE 4:**
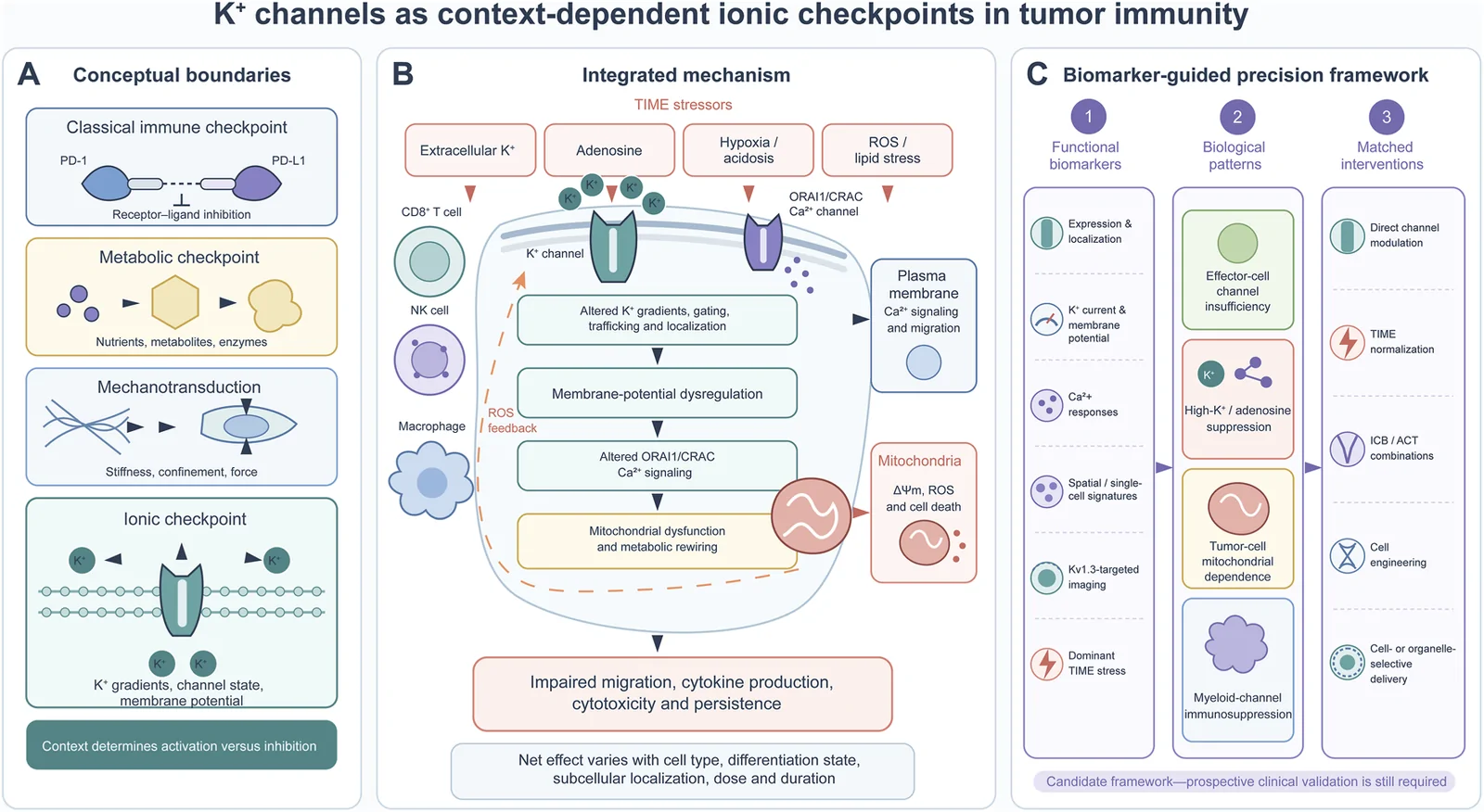
Integrated conceptual, biomarker, and therapeutic framework for K^+^ channel-dependent ionic checkpoints in tumor immunity. **(A)** Classical immune checkpoints are initiated mainly by inhibitory receptor–ligand interactions, metabolic checkpoints by nutrient and metabolite availability, and mechanotransduction by physical properties of the tissue. In contrast, an ionic checkpoint is defined by ion gradients, channel gating and trafficking, membrane potential, and ion-dependent signaling as proximal regulatory variables. The direction of its effect depends on cell type, differentiation state, subcellular localization, microenvironmental context, dose, and duration. **(B)** Extracellular K^+^ accumulation, adenosine, hypoxia, acidosis, oxidative stress, and lipid stress alter K^+^ gradients and channel functional states, thereby disrupting membrane potential, ORAI1/CRAC-mediated Ca^2+^ entry, mitochondrial homeostasis, and metabolic adaptation. Mitochondrial dysfunction and ROS can, in turn, modify channel activity and trafficking, creating a bidirectional feedback loop that influences immune-cell migration, cytokine production, cytotoxicity, and persistence. **(C)** Translation requires integration of channel expression, localization, current, Ca^2+^ responses, spatial context, and dominant TIME stress to stratify patients and select among direct channel modulation, upstream TIME normalization, combination immunotherapy, cell engineering, and cell- or organelle-selective delivery.

Therapeutic strategies informed by ionic-checkpoint biology can be divided into direct modulation of channel activity or subcellular channel pools and indirect restoration of channel-supported immune function by alleviating upstream TIME stress. Table 1 summarizes representative strategies, their principal cellular or subcellular contexts, levels of evidence, and key translational limitations. Figure 3 compares the regulatory levels targeted by classical immune checkpoints and K^+^ channel-dependent ionic-checkpoint regulation, whereas Figure 4 integrates the conceptual boundaries, functional biomarkers, patient-stratification framework, and matched therapeutic options proposed in this review.

**TABLE 1:** Representative direct and indirect therapeutic strategies related to K^+^ channels and ionic-checkpoint regulation in cancer.

Strategy class	Target/pathway	Representative intervention(s)	Primary cellular or compartmental context	Immune-relevant mechanism or therapeutic rationale	Evidence and translational status	Key limitation	References
Direct channel modulation	KCa3.1 activation	1-EBIO; NS309; SKA-31	CD8^+^ T cells and NK cells	Restores KCa3.1-supported hyperpolarization, Ca^2+^ entry, chemotaxis and cytotoxicity; increases intratumoral granzyme B^+^ NK cells	Patient-derived cells; humanized HNSCC mouse model; preclinical	Effects are cell- and pH-dependent; systemic vascular and tumor-cell KCa3.1 activation must be controlled	(Chimote et al., 2018; Cozzolino and Panyi, 2024; Alshwimi et al., 2025)
Direct channel modulation	Kv1.3 and KCa3.1 inhibition	Vm24; TRAM-34	CD19-specific CEM-CAR cells and primary CAR-T cells	Context-specific channel blockade increased tumor-cell killing efficiency in 3D spheroids without increasing CAR-T infiltration	*In vitro* monolayer and 3D spheroid models; no *in vivo* validation	May not generalize to native T/NK cells, in which blockade can impair migration or signaling; direction of benefit is context-dependent	(Medyouni et al., 2024; Jusztus et al., 2025)
Direct channel modulation	KCa3.1 inhibition	TRAM-34	Glioma-infiltrating microglia/macrophages and glioma cells	Suppresses KCa3.1-dependent protumor microglial/macrophage programs and alters TAM morphology, recruitment and phagocytic behavior	*In vitro*, *ex vivo* and syngeneic mouse glioma studies; preclinical	Tumor-cell and myeloid-cell effects are difficult to separate; morphological change alone does not establish functional reprogramming	(Grimaldi et al., 2016; Massenzio et al., 2022)
Organelle-directed channel targeting	Mitochondrial Kv1.3	PAPTP; PCARBTP	Tumor-cell mitochondria	Induces ROS accumulation, mitochondrial depolarization and apoptosis; can sensitize pancreatic tumors to chemotherapy	Cellular and immunocompetent orthotopic mouse models; preclinical	Immune restoration is indirect and incompletely resolved; mitochondrial delivery, tissue selectivity and long-term safety remain concerns	(Li et al., 2022a; Patel et al., 2023)
Organelle-directed channel targeting	Mitochondrial KCa3.1	MitoKCa3.1-targeting small molecules	Tumor-cell mitochondria	Triggers mitochondrial dysfunction and tumor-cell death, reducing tumor growth and metastasis *in vivo*	Cellular and mouse tumor/metastasis models; preclinical	Molecular selectivity and effects on immune-cell mitochondrial KCa3.1 require further definition	(Bachmann et al., 2022)
Direct channel modulation	KCa3.1/*KCNN4*	Senicapoc, with or without perampanel	Glioblastoma cells and brain tumor microenvironment	Tests whether pharmacological KCa3.1 inhibition, alone or with AMPA-receptor blockade, produces intratumoral pharmacodynamic effects	SENIPERA randomized window-of-opportunity Phase 0/1 trial (NCT07284069); protocol published	Designed primarily for safety, tumor exposure and biological activity; immune benefit and clinical efficacy are not yet established	(Eschen et al., 2026)
Channel-directed biologics	Kv10.1 or KCNK9/TASK-3 surface protein	Anti-Kv10.1 scFv/nanobody-TRAIL; anti-KCNK9 antibody Y4	Channel-high tumor cells	Uses abnormal surface channel expression for tumor-selective ligand delivery or antibody-induced internalization rather than broad current blockade	Cellular and mouse xenograft/metastasis studies; preclinical	Direct immune mechanisms are unproven; efficacy depends on stable and sufficiently selective tumor-surface expression	(Hartung and Pardo, 2016; Sun et al., 2016; Hartung et al., 2020)
Direct channel modulation	Kir2.1/*Kcnj2*	Myeloid-specific *Kcnj2* deletion; pharmacological Kir2.1 inhibition	TAMs in high-K^+^ tumor regions	Reduces electrochemical-gradient-dependent glutamine uptake, shifts metabolism from oxidative phosphorylation toward glycolysis and promotes an inflammatory antitumor macrophage state, thereby limiting tumor growth	Myeloid-specific conditional knockout, pharmacological inhibition, syngeneic mouse tumors and patient-derived xenograft models; preclinical	Clinically deployable Kir2.1-selective inhibitors and myeloid-directed delivery systems remain unavailable; systemic inhibition may affect Kir2.1-dependent functions in excitable tissues	(Chen et al., 2022)
Direct channel modulation	Kir4.1/*Kcnj10*	Pharmacological Kir4.1 inhibition, alone or combined with anti-PD-1 therapy	MDSCs in high-K^+^ tumor regions	Suppresses FABP3-dependent fatty-acid uptake and oxidation, reduces MDSC-mediated inhibition of CD8^+^ T-cell proliferation and IFN-γ production, and improves responsiveness to anti-PD-1 therapy	*In vitro* and *ex vivo* immune assays and mouse tumor models; preclinical	Selective clinically applicable inhibitors and MDSC-targeted delivery systems are lacking; potential effects on neural and renal Kir4.1 require evaluation	(Xie et al., 2026)
Indirect restoration of channel-supported immunity	CD39/extracellular ATP-adenosine axis	IPH5201	Immune cells, tumor cells, stroma and vasculature in the TIME	Preserves immunostimulatory extracellular ATP and limits downstream adenosine production, supporting antitumor immune activation	First-in-human Phase I monotherapy/combination study; intratumoral CD39 activity was reduced	Clinical antitumor activity was limited mainly to disease stabilization; effects on K^+^ channel function were not measured directly	(Powderly et al., 2025)
Indirect restoration of channel-supported immunity	CD73/adenosine production	Oleclumab; quemliclustat	Immune cells and adenosine-rich TIME	Reduces extracellular adenosine, thereby relieving A_2_A/A_2_B receptor-mediated suppression of T/NK-cell function and KCa3.1-supported motility	Oleclumab: Phase I and randomized Phase Ib/II studies. Quemliclustat: randomized Phase Ib ARC-8; Phase III validation ongoing	Clinical signals vary by tumor type and regimen; early-phase and nonconcurrent-control data do not establish definitive efficacy	(Bendell et al., 2023; Coveler et al., 2024; Wainberg et al., 2026)
Indirect restoration of channel-supported immunity	A_2_A adenosine receptor	AZD4635; ciforadenant	T cells and other immune cells in the TIME	Blocks A_2_AR-cAMP-PKA immunosuppression, with the rationale of restoring Ca^2+^ signaling, migration and effector function	Phase I/II clinical studies in solid tumors, including mCRPC and RCC.	Responses have been inconsistent; pathway activity, combination partners and patient selection strongly influence benefit	(Fong et al., 2019; Lim et al., 2022; Falchook et al., 2024)
Indirect restoration of channel-supported immunity	A_2_A and A_2_B adenosine receptors	Etrumadenant	Immune and stromal compartments in the TIME	Dual receptor blockade limits adenosine-mediated suppression across immune and stromal cells and may complement PD-1 and VEGF-directed therapy	Randomized Phase II ARC-9 study in previously treated metastatic colorectal cancer	The active regimen also contained zimberelimab, mFOLFOX6 and bevacizumab; efficacy cannot be attributed to etrumadenant alone	(Cecchini et al., 2026)
Indirect normalization of the TIME	Hypoxia/acidosis and carbonic anhydrase IX	CAIX inhibition; pH-normalizing approaches	Hypoxic/acidic tumor regions and infiltrating lymphocytes	Reduces extracellular acidosis and hypoxia-linked stress that can impair channel responsiveness, Ca^2+^ signaling and immune-cell function	Mechanistic and mouse combination-immunotherapy studies; predominantly preclinical	The channel link is indirect; spatial heterogeneity and systemic acid-base effects complicate dosing and biomarker selection	(Chafe et al., 2019; Navarro et al., 2022; Cozzolino and Panyi, 2024)

### 5.1 Direct channel-targeting strategies

Direct channel interventions include channel inhibition, pharmacological activation, subcellular targeting, and antibody-mediated delivery. Most existing studies, however, have focused on tumor-cell proliferation, migration, invasion, apoptosis, and drug resistance as endpoints (Xia et al., 2023). In effector lymphocytes, the consequences of Kv1.3 and KCa3.1 modulation are strongly cell-type and function dependent. CD8^+^ T cells from patients with HNSCC exhibit a KCa3.1 functional defect, and pharmacological KCa3.1 activation partially restores chemotactic responses in an adenosine-rich environment (Chimote et al., 2018). More recent work showed that the KCa3.1 activator SKA-31 enhances chemotaxis and cytotoxicity in human NK cells and increases tumor infiltration by total and granzyme B^+^ NK cells in a humanized HNSCC model. Conversely, KCa3.1 blockade with TRAM-34 inhibits NK-cell chemotaxis (Alshwimi et al., 2025).

Channel inhibition does not produce uniform effects across effector-cell contexts. In CAR-T cells and three-dimensional tumor-spheroid models, inhibition of Kv1.3 or KCa3.1 with Vm24 or TRAM-34 increases target-cell clearance without substantially changing CAR-T-cell infiltration (Medyouni et al., 2024; Jusztus et al., 2025). Further work showed that Kv1.3 is recruited to the immunological synapse together with the CAR, whereas restricting Kv1.3 lateral trafficking reduces early target-cell clearance (Medyouni et al., 2025). These findings indicate that suppressing channel conductance, disrupting spatial channel organization, and altering the duration of Ca^2+^ signaling are not equivalent interventions. K^+^ channel activation may favor migration and sustained Ca^2+^ entry, whereas a moderate reduction in current may, in some CAR-T models, maintain cytosolic Ca^2+^ within a range more favorable for degranulation and target-cell killing.

KCa3.1 is also a representative target for direct modulation of TAM states. In glioma models, KCa3.1 inhibition with TRAM-34 reduces protumor features in tumor-associated microglia and macrophages, enhances inflammatory phenotypes, and limits tumor growth (Grimaldi et al., 2016). Intravital two-photon imaging further showed that KCa3.1 inhibition rapidly changes TAM morphology and process architecture within the tumor core, suggesting that the channel contributes to maintenance of a dynamic TAM state (Massenzio et al., 2022). Morphological changes alone, however, do not demonstrate functional reprogramming. In an immunocompetent glioma model, TRAM-34 combined with radiotherapy suppressed postirradiation tumor invasion and prolonged survival, but did not produce a clearly detectable change in immune-cell composition (Stransky et al., 2023).

Subcellular targeting offers a therapeutic route distinct from modulation of plasma-membrane channels. The mitochondria-targeted compounds PAPTP and PCARBTP accumulate in mitochondria and inhibit mitoKv1.3, disrupting mitochondrial function and enhancing chemotherapy efficacy in pancreatic ductal adenocarcinoma models (Li W. et al., 2022). MitoTRAM-34 and rev-mitoTRAM target mitoKCa3.1, increase mtROS, decrease mitochondrial membrane potential, and fragment the mitochondrial network, ultimately inducing tumor-cell death and reducing tumor growth and metastasis (Bachmann et al., 2022).

Antibodies and nanobodies can exploit extracellular channel structures to improve targeting selectivity. The VHH-D9-scTRAIL fusion protein, which combines an anti-Kv10.1 nanobody with single-chain TRAIL, concentrates apoptotic signaling in Kv10.1-positive tumor cells and enhances apoptosis in cell cultures and three-dimensional tumor spheroids (Hartung et al., 2020). The Y4 monoclonal antibody directed against the extracellular domain of KCNK9 induces channel internalization, reduces tumor-cell viability, and suppresses tumor growth and metastasis in mice (Sun et al., 2016).

Emerging delivery and engineering technologies may help overcome the limited cell-type and subcellular selectivity of conventional channel modulators. Nanoparticles, lipid nanoparticles, antibody- or nanobody-guided carriers, and stimulus-responsive systems could enrich small molecules or RNA cargoes in defined tumor or immune-cell populations. Extracellular channel-directed targeting is supported by the anti-Kv10.1 VHH-D9-scTRAIL fusion protein and the KCNK9-directed Y4 monoclonal antibody, which respectively concentrate apoptotic signaling in channel-positive tumor cells and induce channel internalization (Sun et al., 2016; Hartung et al., 2020). These studies do not establish selective manipulation of immune-cell Kv1.3 or KCa3.1 in patients, but they show that extracellular channel epitopes can serve as targeting handles.

RNA therapeutics and CRISPR-based engineering provide complementary approaches when pharmacological selectivity is insufficient. *Ex vivo* editing is especially attractive for ACT because the intended immune-cell population can be modified before reinfusion; clustered regularly interspaced short palindromic repeats/CRISPR-associated protein 9 (CRISPR/Cas9)-mediated deletion of ADORA2A enhances CAR-T-cell function in adenosine-rich solid-tumor models (Giuffrida et al., 2021). Analogous editing of channel abundance, trafficking regulators, or TIME-sensing pathways is feasible in principle but requires careful control to avoid excessive Ca^2+^ signaling, altered differentiation, or impaired persistence. Induced-proximity channel downregulation is another emerging platform. Recruitment of endogenous NEDD4-2 with divalent nanobodies reduced the surface density and function of CaV2.2, KCNQ1, and ENaC in heterologous systems (Darko-Boateng et al., 2025). This result is proof of principle for targeted ion-channel downregulation, not evidence for Kv1.3- or KCa3.1-directed tumor immunotherapy. Classical proteolysis-targeting chimera (PROTAC) terminology should therefore be used cautiously for this platform.

### 5.2 Indirect restoration of K^+^ channel-supported antitumor immune function

Unlike direct K^+^ channel modulation, indirect strategies aim to restore channel-supported immune-cell function by attenuating upstream suppressive signals in the TIME. Cellular injury and treatment-induced tumor-cell death release extracellular ATP. CD39 sequentially hydrolyzes ATP and ADP to AMP, and CD73 then converts AMP to adenosine. CD39 inhibition preserves immunostimulatory extracellular ATP and reduces the supply of AMP, whereas CD73 inhibition blocks the terminal step of adenosine production. The resulting adenosine predominantly activates cAMP/PKA signaling through adenosine A2A receptor (A_2_AR) and adenosine A2B receptor (A_2_BR) on immune cells, suppressing T- and NK-cell migration, cytokine release, and cytotoxicity. Blocking CD39, CD73, or adenosine receptors can therefore relieve metabolic suppression upstream of K^+^ channels, although these interventions do not directly target channel proteins (Perrot et al., 2019; Allard et al., 2020; Powderly et al., 2025).

A_2_AR activation selectively reduces KCa3.1 currents through cAMP and PKA-I, with little effect on Kv1.3 currents. KCa3.1 inhibition compromises maintenance of membrane potential, reduces Ca^2+^ entry, and limits T-cell migration and IL-2 release (Chimote et al., 2013). In CD8^+^ T cells from patients with HNSCC, reduced KCa3.1 activity further increases sensitivity to adenosine, whereas pharmacological enhancement of KCa3.1 partially restores chemotaxis in adenosine-rich conditions (Chimote et al., 2018). Adenosine also suppresses NK-cell maturation, IFN-γ production, degranulation, and target-cell killing (Chambers et al., 2018; Young et al., 2018).

Clinical development of adenosine-axis interventions has progressed from target-engagement and pharmacodynamic studies toward efficacy evaluation. CD39 inhibition remains at an early clinical stage. Anti-CD39 antibodies have been shown to reduce intratumoral CD39 enzymatic activity with generally manageable safety, but the antitumor effects observed thus far have largely been limited to disease stabilization (Powderly et al., 2025). Clinical evidence for CD73 inhibition is more extensive: oleclumab and quemliclustat have shown acceptable safety, target-related pharmacodynamic changes, and signals of disease control or survival benefit in several solid tumors. Outcomes, however, vary across tumor types and combination regimens; some studies have not demonstrated clear survival benefits, and early-phase or nonconcurrent-control designs limit efficacy interpretation. Relevant agents, trial stages, patient populations, and principal findings are summarized in Table 2.

**TABLE 2:** Clinical development of direct K^+^ channel-targeting and upstream ionic-checkpoint interventions in cancer.

Intervention	Target/strategy type	Representative trial(s)	Phase/status	Indication/regimen	Main finding or development objective and translational interpretation	References
Senicapoc	KCa3.1/*KCNN4* inhibition; direct K^+^ channel targeting	SENIPERA; NCT07284069	Early Phase I (Phase 0/1); recruiting	Newly diagnosed glioblastoma; senicapoc alone or with perampanel in a window-of-opportunity design	Assesses tolerability, dose, tumor/brain exposure and pharmacodynamic effects; no cancer efficacy result is available. Immune effects remain exploratory and cannot be separated from direct glioma-cell or neural effects	(Eschen et al., 2026)
IPH5201	CD39 blockade; upstream ATP–adenosine-axis intervention	First-in-human Phase I; MATISSE, NCT05742607	Phase I completed; Phase II recruiting	Advanced solid tumors and resectable stage II–IIIA NSCLC; alone or combined with durvalumab ± platinum chemotherapy	Phase I showed target engagement and reduced intratumoral CD39 activity but limited antitumor activity. Interim MATISSE pCR findings are single-arm and do not establish comparative efficacy or direct K^+^ channel recovery	(Powderly et al., 2025; Barlesi et al., 2026)
Oleclumab	CD73 inhibition; upstream adenosine-production blockade	NCT02503774; COAST, NCT03822351; PACIFIC-9, NCT05221840	Phase I/II completed; Phase III active, not recruiting	Advanced solid tumors and unresectable stage III NSCLC; oleclumab ± durvalumab after cCRT	COAST showed numerically higher ORR and longer PFS with durvalumab plus oleclumab, without a nominally significant OS association. PACIFIC-9 is the confirmatory Phase III study	(Bendell et al., 2023; Barlesi et al., 2024; Coveler et al., 2024; Aggarwal et al., 2025)
Quemliclustat	CD73 inhibition; upstream adenosine-production blockade	ARC-8, NCT04104672; PRISM-1, NCT06608927	Phase Ib reported; Phase III active, not recruiting	Treatment-naive metastatic PDAC; quemliclustat plus gemcitabine/nab-paclitaxel, with or without zimberelimab	ARC-8 reported a survival signal, reduced adenosine-regulated NR4A expression and T-cell activation. Its early-phase design and nonconcurrent comparisons preclude definitive efficacy claims; PRISM-1 provides randomized validation	(Wainberg et al., 2026)
AZD4635	A_2_AR antagonism; upstream receptor blockade	NCT02740985; NCT04089553; NCT04495179	Phase I/II completed or reported	Advanced solid tumors and mCRPC; monotherapy or combinations with durvalumab, oleclumab and/or cabazitaxel	Phase I established pharmacodynamic activity and manageable safety, whereas later mCRPC combinations showed limited antitumor activity. This illustrates that mechanistic rationale does not ensure clinical benefit	(Lim et al., 2022; Falchook et al., 2024)
Ciforadenant	A_2_AR antagonism; upstream receptor blockade	NCT02655822; NCT05501054	Phase I/Ib reported; Phase Ib/II ongoing	Refractory or first-line clear-cell RCC; alone or with atezolizumab, or with ipilimumab plus nivolumab	Human studies showed increased CD8^+^ T-cell infiltration, TCR-clonal expansion and preliminary activity. Comparative efficacy and the contribution of each combination component remain unresolved	(Fong et al., 2019)
Etrumadenant	Dual A_2_AR/A_2_BR antagonism; upstream receptor blockade	ARC-9, NCT04660812	Randomized Phase II reported	Previously treated metastatic colorectal cancer; etrumadenant plus zimberelimab, mFOLFOX6 and bevacizumab versus regorafenib	ARC-9 reported longer PFS and OS for the etrumadenant-containing regimen. Because the experimental arm was multi-agent, the observed benefit cannot be attributed to etrumadenant alone	(Cecchini et al., 2026)

Adenosine-receptor blockade has likewise generated clinical mechanistic evidence. The A_2_AR antagonists AZD4635 and ciforadenant modulate adenosine-associated immunosuppression and have been associated with tumor control, T-cell infiltration, or TCR clonal expansion in subsets of patients, although overall clinical responses have been inconsistent. A multi-agent regimen containing the dual A_2_AR/A_2_BR antagonist etrumadenant improved progression-free and overall survival in metastatic colorectal cancer. Because the experimental regimen also included immune-checkpoint blockade, chemotherapy, and antiangiogenic therapy, the observed benefit cannot be attributed to etrumadenant alone (Fong et al., 2019; Lim et al., 2022; Falchook et al., 2024; Cecchini et al., 2026). Beyond the adenosine pathway, alleviating hypoxia, acidosis, and nutrient-metabolic stress may indirectly improve channel-supported immune function. Interventions that reduce tumor hypoxia can increase T-cell infiltration and improve responses to immunotherapy, whereas carbonic anhydrase IX inhibition or buffering of tumor acidity improves T-cell function and ICB efficacy in experimental models (Jayaprakash et al., 2018; Chafe et al., 2019). Overall, clinical development of upstream adenosine-axis interventions is more advanced than that of direct K^+^ channel modulation, but their independent therapeutic contributions, responsive tumor types, and predictive biomarkers require further evaluation in randomized controlled trials.

### 5.3 Combination therapy and translational challenges

Classical immune checkpoints and K^+^ channel-dependent ionic regulation act at distinct but interdependent levels. PD-1/PD-L1 and CTLA-4 principally constrain receptor-proximal activation and costimulation, whereas ionic-checkpoint states determine whether membrane potential, Ca^2+^ entry, migration, and metabolic adaptation remain sufficient after receptor inhibition is relieved. Ionic-checkpoint interventions should therefore not be positioned as substitutes for ICB; their most plausible role is to restore a demonstrated electrophysiological or microenvironmental defect that limits ICB efficacy.

Patient-derived samples provide preliminary support for this mechanistic complementarity. In patients with HNSCC, PD-1 blockade increases Kv1.3 and KCa3.1 currents in cytotoxic T cells and is accompanied by improved Ca^2+^ signaling and chemotactic capacity; the increase in Kv1.3 activity is more pronounced among clinical responders (Newton et al., 2020; Gawali et al., 2021). Existing studies, however, are based mainly on pre- and post-treatment comparisons of peripheral-blood cells or *ex vivo* functional assays and do not demonstrate that combining K^+^ channel modulators with ICB improves clinical outcomes. The PD-1/PD-L1 axis is currently the only classical checkpoint pathway supported by patient-derived electrophysiological evidence. Associations of LAG-3, TIM-3, and TIGIT with K^+^ channels derive mainly from studies of adenosine signaling, coinhibitory-receptor expression, or combination therapy and should not be interpreted as evidence of direct channel regulation.

Compared with direct channel-targeting drugs, relief of adenosine-mediated suppression in the TIME has a stronger rationale for combination therapy. In murine solid-tumor models, A_2_AR inhibition reduces expression of coinhibitory receptors including PD-1, LAG-3, and TIM-3 and enhances the antitumor effects of checkpoint blockade and adoptive T-cell therapy (Leone et al., 2018). Combined inhibition of A_2_AR and TIM-3 further improves cytoskeletal polarization and cytotoxicity in tumor spheroids and murine tumor models, suggesting that adenosine signaling and TIM-3 impose complementary constraints at the effector phase (Edmunds et al., 2022). Adenosine-axis inhibitors have entered clinical development in combination with ICB, but outcomes remain inconsistent. Some CD73 or A_2_AR/A_2_BR inhibitors combined with PD-L1 blockade have produced signals of disease control or progression-free survival benefit, whereas other studies have shown only limited activity (Fong et al., 2019; Falchook et al., 2024; Aggarwal et al., 2025; Cecchini et al., 2026). Relief of TIME-mediated suppression therefore does not necessarily translate into consistent clinical benefit. Differences in adenosine production, receptor expression, immune infiltration, and other metabolic pressures across tumors may jointly determine treatment response. Moreover, multi-agent trials generally cannot resolve the independent contributions of adenosine-axis intervention, ICB, and other treatment components.

Ionic-checkpoint interventions may also improve ACT, but available studies demonstrate substantial context dependence. High extracellular K^+^ suppresses T-cell effector programs in murine and human tumor samples, whereas enhancing K^+^ efflux from T cells improves ACT efficacy in mice (Eil et al., 2016). Conversely, K^+^ accumulation can restrict effector differentiation while preserving stem cell-like features, suggesting that different ionic-modulation strategies may be required during cell manufacturing and during the intratumoral effector phase (Vodnala et al., 2019). Recent three-dimensional tumor-spheroid studies found that brief inhibition of Kv1.3 or KCa3.1 increases target-cell clearance by some CAR-T cells. These findings remain limited to *in vitro* models and do not establish that prolonged or systemic channel blockade benefits CAR-T-cell therapy (Medyouni et al., 2024; Jusztus et al., 2025). By contrast, genetic deletion of A_2_AR enhances CAR-T-cell effector function and therapeutic activity in murine solid-tumor models, providing stronger preclinical support for engineering cell-intrinsic resistance to the TIME (Giuffrida et al., 2021).

NK-cell therapies may likewise be jointly constrained by adenosine and channel function. A_2_AR signaling suppresses NK-cell maturation and antitumor activity, whereas CD73 targeting enhances chimeric antigen receptor natural killer (CAR-NK)-cell tumor homing, degranulation, IFN-γ production, and cytotoxicity *in vitro* and in a lung cancer xenograft model (Wang et al., 2018; Young et al., 2018). These findings support evaluation of CAR-NK therapy combined with adenosine-axis intervention in CD73-high solid tumors, although current evidence remains predominantly cellular and preclinical.

Beyond ICB and ACT, ionic-checkpoint interventions could be incorporated into broader multimodal immunotherapy, but the strength of evidence differs substantially among combinations. Relatively direct preclinical support exists for combining adenosine-pathway modulation with CAR-T or CAR-NK therapy and for combining KCa3.1 inhibition with radiotherapy in glioma models (Giuffrida et al., 2021; Stransky et al., 2023). Bispecific antibodies, oncolytic viruses, STING agonists, and engineered IL-2 or IL-15 variants may increase immune-cell recruitment or activation and thereby increase the requirement for sustained membrane potential, Ca^2+^ signaling, and metabolic adaptation. Macrophage-directed strategies may also be combined with Kir2.1-, Kir4.1-, or KCa3.1-associated interventions to reprogram suppressive myeloid states. These combinations are mechanistically plausible, but direct evidence that they synergize with K^+^ channel modulation remains limited. Future studies should compare channel- or TIME-directed combinations against each component alone and should include immune-cell-specific functional readouts rather than relying exclusively on tumor-volume reduction.

The slow clinical translation of direct K^+^ channel targeting reflects several interacting barriers. First, many channel modulators lack sufficient subtype selectivity, whereas Kv1.3, KCa3.1, and related channels are expressed across tumor cells, effector lymphocytes, myeloid cells, and normal cardiovascular or nervous tissues. Second, the same channel can exert opposing effects in different cells or subcellular compartments: plasma-membrane Kv1.3 and KCa3.1 support Ca^2+^ signaling and migration in effector lymphocytes, whereas inhibition of mitochondrial channel pools in tumor cells can promote mitochondrial depolarization, ROS accumulation, and cell death. Third, functional redundancy and compensatory remodeling among K^+^ channel families may reduce the durability of single-channel intervention. Fourth, systemic exposure, inadequate tumor penetration, short intratumoral retention, and simultaneous access to plasma-membrane and mitochondrial channel pools create pharmacokinetic and pharmacodynamic uncertainty. Finally, interpatient variation in channel expression, membrane localization, immune-cell composition, extracellular K^+^, adenosine, hypoxia, and acidity makes a uniform treatment strategy unlikely to succeed. These limitations are compounded by the absence of validated target-occupancy assays and pharmacodynamic thresholds for channel current, Ca^2+^ recovery, or metabolic restoration (Alexander et al., 2023). Clinical development will therefore require cell- or organelle-selective delivery, functional patient stratification, and early incorporation of pharmacodynamic biomarkers.

### 5.4 Functional biomarkers and precision patient stratification

The translational relevance of K^+^ channels extends beyond their use as therapeutic targets. Channel-related measurements could potentially serve as prognostic, predictive, pharmacodynamic, or imaging biomarkers, although none has yet achieved prospective clinical validation. Prognostic analyses may assess whether channel abundance, localization, or functional state is associated with immune infiltration, recurrence, or survival. Predictive biomarkers should identify patients in whom channel dysfunction or an upstream TIME stress is likely to constrain ICB or ACT. Pharmacodynamic biomarkers should demonstrate that an intervention has altered the intended pathway, for example, through changes in surface channel density, K^+^ current, membrane potential, Ca^2+^ flux, migration, cytokine production, or metabolic recovery. These categories should not be conflated: an association between high channel expression and survival does not establish that the channel predicts treatment response or represents a therapeutically actionable dependency.

Current evidence is strongest for Kv1.3-associated functional and imaging readouts but remains preliminary. In patients with HNSCC receiving neoadjuvant pembrolizumab, recovery of Kv1.3 and KCa3.1 currents was accompanied by improved Ca^2+^ signaling and migration, and the increase in Kv1.3 activity was more pronounced among clinical responders (Newton et al., 2020; Gawali et al., 2021). These observations support investigation of channel current as a pharmacodynamic or predictive biomarker but do not define a validated clinical cutoff. In the CT26 murine colon cancer model, uptake of a Kv1.3-targeted positron emission tomography (PET) tracer reflected accumulation of Kv1.3-expressing effector-memory T cells and distinguished immunotherapy-responsive from nonresponsive tumors (Goggi et al., 2022). This provides proof of concept for noninvasive imaging of a channel-associated immune population; however, specificity, reproducibility, and predictive performance require validation in additional tumor models and prospective human studies.

Single-cell and spatial technologies could support biomarker discovery by assigning channel-associated signatures to specific immune and tumor-cell populations. Single-cell RNA sequencing can distinguish channel-gene expression among effector, memory, exhausted, and dysfunctional T-cell states, whereas spatial transcriptomics can determine whether these populations are located within tumor nests, stromal regions, perivascular niches, invasive margins, or necrosis-adjacent areas. Multiplex immunofluorescence and imaging mass cytometry can further assess channel protein together with immune-lineage, activation, exhaustion, metabolic, and vascular markers. Spatially resolved studies have already shown that tumors contain structured cellular neighborhoods and multicellular immune hubs that cannot be reconstructed from bulk measurements alone (Jackson et al., 2020; Pelka et al., 2021). Such measurements should be integrated with electrophysiology and functional imaging because mRNA abundance cannot establish membrane insertion, gating, current density, or organelle localization. A channel-associated multi-omics signature should therefore be considered a biomarker candidate rather than a direct substitute for channel function.

A precision-medicine framework should integrate the cellular source and functional state of the channel with the dominant suppressive ecology of the tumor. Patients could provisionally be stratified into four nonexclusive biological patterns: effector-cell channel insufficiency, in which inadequate plasma-membrane channel function limits Ca^2+^ signaling or migration; high-K^+^- or adenosine-dominant suppression, in which the upstream microenvironmental stress is the more appropriate target; tumor-cell mitochondrial-channel dependence, in which organelle-directed inhibition may preferentially induce tumor-cell death; and myeloid-channel-dependent immunosuppression, in which Kir2.1, Kir4.1, or KCa3.1 supports suppressive TAM or MDSC states. Assignment to these categories should integrate channel gene and protein expression, cell identity, plasma-membrane versus mitochondrial localization, current density, membrane potential, Ca^2+^ responses, extracellular K^+^, adenosine-pathway activity, hypoxia, acidity, and prior treatment. Prospective studies are required to determine whether such integrated profiles can function as companion diagnostics or predict benefit from channel-directed, TIME-directed, or combination therapy.

## 6 Discussion and future perspectives

### 6.1 Unresolved controversies and boundary conditions

Several unresolved controversies limit the formulation of a universal therapeutic rule for K^+^ channels in tumor immunity. The first is whether a channel should be activated or inhibited. Kv1.3 and KCa3.1 activity can support membrane polarization, Ca^2+^ entry, migration, and sustained effector function in conventional T and NK cells. Conversely, brief pharmacological inhibition of these channels has increased target-cell clearance in some CAR-T-cell and three-dimensional tumor-spheroid models (Medyouni et al., 2024; Jusztus et al., 2025). These findings are not necessarily contradictory because channel conductance, synaptic localization, Ca^2+^-signal amplitude, exposure duration, and differentiation state are distinct variables. Moderate or transient current reduction may preserve a Ca^2+^ range favorable for degranulation in one CAR-T-cell configuration, whereas sustained or systemic inhibition may compromise migration, proliferation, or persistence.

A second controversy concerns the effects of extracellular and intracellular K^+^. Elevated extracellular K^+^ suppresses acute effector programs and tumor-cell killing, whereas prolonged K^+^-associated signaling can preserve stem cell-like features and the developmental potential of T cells (Eil et al., 2016; Vodnala et al., 2019). Thus, an ionic state that limits immediate cytotoxicity may have different consequences during *ex vivo* cell manufacturing, early differentiation, intratumoral effector activity, or memory formation. The therapeutic objective may therefore need to change over time: preservation of less-differentiated cells during manufacturing should not be assumed to require the same ionic conditions as rapid tumor-cell killing after infusion.

A third boundary condition is subcellular localization. Plasma-membrane channels regulate membrane potential and Ca^2+^ entry, whereas mitochondrial Kv1.3 and KCa3.1 participate in regulation of ΔΨm, ROS production, and apoptosis (Bachmann et al., 2022; Li W. et al., 2022). Inhibition of a mitochondrial channel pool in tumor cells may be therapeutically beneficial even when inhibition of the corresponding plasma-membrane channel impairs immune-cell function. Pharmacological inhibitors, acute electrophysiological blockade, long-term genetic deletion, and disruption of channel trafficking are therefore not equivalent experimental interventions. Conclusions may also vary with species, tumor model, immune-cell subset, drug concentration, treatment duration, and whether tumor cells and immune cells are examined separately or in coculture.

### 6.2 Experimental and translational roadmap

Resolving these controversies will require inducible, cell-type-specific, and compartment-aware experimental designs. Channel activity should be manipulated separately in tumor cells, T-cell and NK-cell subsets, DCs, macrophages, and MDSCs, with tumor-cell monocultures, immune-cell monocultures, and cocultures included as parallel controls. Patient-derived immune cells should be integrated with autologous tumor organoids, tumor-tissue fragments, and three-dimensional TIME models. Surface channel density, K^+^ current, membrane potential, and Ca^2+^ flux should be measured together with migration, cytokine release, cytotoxicity, proliferation, exhaustion, and persistence. Studies of mitochondrial channels should additionally measure ΔΨm, oxidative phosphorylation, ATP production, ROS, substrate utilization, mitochondrial morphology, and quality-control pathways.

Clinical translation should proceed through biomarker-enriched, mechanism-driven studies rather than unselected systemic administration of broad channel modulators. Early trials should establish intratumoral exposure, target occupancy, cell-specific pharmacodynamic effects, and the relationship between channel modulation and immune function before relying on conventional response endpoints. Multiregion and paired pre- and post-treatment sampling should be combined with single-cell, spatial, electrophysiological, and metabolic measurements. Local administration, immune-cell-selective carriers, activation-state-dependent release, mitochondria-targeted delivery, and *ex vivo* engineering may widen the therapeutic window. Ultimately, the most informative clinical question is not whether a particular K^+^ channel is globally activated or inhibited, but whether a defined intervention corrects a demonstrated functional defect in the intended cell population, subcellular compartment, and tumor context.

## 7 Conclusion

K^+^ channels constitute a context-dependent ionic checkpoint that links physicochemical stress in the TIME to immune-cell signaling and function. Unlike classical receptor–ligand checkpoints, this regulatory state is defined by K^+^ gradients, channel gating and trafficking, membrane potential, Ca^2+^ entry, and subcellular channel localization. Kv1.3, KCa3.1, Kir, and K2P channels contribute to T- and NK-cell effector activity, DC-mediated T-cell priming, immune-cell migration, and myeloid immunosuppression, but their effects vary across cell types, tumor regions, treatment stages, and plasma-membrane versus mitochondrial compartments. Consequently, neither universal channel activation nor universal channel inhibition represents an appropriate therapeutic strategy. Direct channel targeting remains limited by insufficient selectivity, systemic toxicity, functional redundancy, pharmacokinetic constraints, and the absence of validated pharmacodynamic biomarkers. Upstream normalization of high extracellular K^+^, adenosine, hypoxia, or acidosis may offer a more feasible route in selected tumors, particularly when combined with ICB or ACT. Translation will require functional and spatial biomarkers, multiregion and longitudinal assessment, and cell- or organelle-selective interventions. As summarized in Figure 4, the therapeutic value of ionic-checkpoint modulation will ultimately depend on correcting a defined electrophysiological defect in the appropriate patient, tumor niche, cellular population, and subcellular compartment.
